# Biotechnological Strategies to Improve Plant Biomass Quality for Bioethanol Production

**DOI:** 10.1155/2017/7824076

**Published:** 2017-08-29

**Authors:** Julián Mario Peña-Castro, Sandra del Moral, Lizeth Núñez-López, Blanca E. Barrera-Figueroa, Lorena Amaya-Delgado

**Affiliations:** ^1^Laboratorio de Biotecnología Vegetal, Instituto de Biotecnología, Universidad del Papaloapan, 68333 Tuxtepec, OAX, Mexico; ^2^Laboratorio de Biología Molecular, Instituto de Biotecnología, Universidad del Papaloapan, 68333 Tuxtepec, OAX, Mexico; ^3^División de Estudios de Posgrado, Universidad del Papaloapan, 68333 Tuxtepec, OAX, Mexico; ^4^Unidad de Biotecnología Industrial, Centro de Investigación y Asistencia en Tecnología y Diseño del Estado de Jalisco, 45019 Zapopan, JAL, Mexico

## Abstract

The transition from an economy dependent on nonrenewable energy sources to one with higher diversity of renewables will not be a simple process. It requires an important research effort to adapt to the dynamics of the changing energy market, sort costly processes, and avoid overlapping with social interest markets such as food and livestock production. In this review, we analyze the desirable traits of raw plant materials for the bioethanol industry and the molecular biotechnology strategies employed to improve them, in either plants already under use (as maize) or proposed species (large grass families). The fundamentals of these applications can be found in the mechanisms by which plants have evolved different pathways to manage carbon resources for reproduction or survival in unexpected conditions. Here, we review the means by which this information can be used to manipulate these mechanisms for commercial uses, including saccharification improvement of starch and cellulose, decrease in cell wall recalcitrance through lignin modification, and increase in plant biomass.

## 1. Introduction

The vast energy demands of modern society are met with fossil fuel such as natural gas, coal, petroleum oil, and its derivatives such as gasoline and diesel. The use of these energy sources has environmental impacts such as air, water, and soil pollution at extraction sites, ducts, and refineries [1], and the anthropogenic mobilization of millions of carbon tons from subsoil to the atmosphere is one of the main factors leading to global warming [2]. In addition, oil-producing countries experience conflicts, long-term economic distortions, and lack of technological diversity associated with fluctuations in the energy market and oil dependence [3, 4]. For example, Mexico, a former leading oil producer, is now a net importer of refined oil from the USA [5]. Gasoline imports have doubled in the last decade creating an inflationary spiral and wide social concerns. According to current economic estimates, this situation will be irreversible for the next decades [6].

Under this scenario, biofuels are proposed as an alternative to fossil fuels, especially ethanol produced as the final fermentation product of natural carbohydrate-consuming yeast, such as* Saccharomyces cerevisiae, Pichia stipitis, *or* Kluyveromyces marxianus*, and bacteria, such as* Zymomonas mobilis *[7]. Mutant and genetically engineered strains of these microorganisms and others (e.g.,* Pichia pastoris *or* Escherichia coli*) have also been proposed to improve ethanol yield through increase in pentose fermentation, improved ethanol tolerance, and use of native starch or cellulose as substrates [7, 8]. Brazil is the pioneer in using bioethanol to fuel transportation activities, followed by the USA. Both countries have 20 and 15 years of experience, respectively, and a combined global production of 90% [9]. Sugarcane juice (*Saccharum* spp.) is the plant raw material in Brazil, whereas corn starch* (Zea mays)* is the plant raw material in the USA. In sugarcane, sucrose is the free carbohydrate easily released by mechanical extraction; in corn starch, since it is a glucose polymer, sugar monomers must be released by heat and enzymatic hydrolysis to make them readily available for fermentation. This process is called saccharification [10, 11].

Over the last decade, cellulose, the most abundant plant polymer in nature, has also been tested as raw material for bioethanol production. It should also undergo saccharification to release its hexose and pentose monomers. Starch is a polymer of low saccharification recalcitrance when compared to cellulose that demands more processing steps because it is interlinked with lignin, a plant polymer composed of aromatic monomers that are difficult to break down. Different mechanical, chemical, and enzymatic processes are needed for cellulose saccharification [11].

The scientific and technological studies on bioethanol production have attracted attention in recent years. Four decades ago, only a few patents of this process were registered; however, in the last decade, hundreds of them are filled [9]. These patents involve all stages of the process, from fermentation bioengineering to microbial strain improvement, engineering of saccharification enzymes, and genetic improvement of plant raw material.

If free carbohydrates, saccharified starch/cellulose, or using a combination for increased efficiency through whole plant biomass use [e.g., [16]] is intended for bioethanol production, improving the content of these molecules in plants bred for the bioethanol industry is needed [17–19]. In the present study, we update the knowledge of plant biotechnology strategies with promising application in the bioethanol industry and discuss the positive impact in our current understanding of carbon allocation in plants.

## 2. Plant Biomass as Raw Material for the Production of Bioethanol

Plant biomass has been used for centuries as an energy source, for example, wood for heating. Given the growing demand of renewable materials used to substitute industrial products, plant biomass is considered a strategic resource for biofuel production, especially bioethanol [20]. Additionally, plant biomass can also be a source of other chemical molecules of interest such as lactate, acetone, furfural, lubricants, and jet fuel [21, 22].

The production of ethanol by fermentation of plant sugars to produce wine, beer, and other alcoholic beverages is a process well recognized by all civilizations. We now know that the substrates of this process are free or polymerized sugars. Each of these molecules has its own* in planta *dynamics of accumulation, compartmentalization, and function [23, 24].

The accumulation of free sugars is highly important in plants, for their role in energy production when used by cells to obtain ATP and NADH through glycolysis. Recently, carbohydrates have also been determined as an indicator of the photosynthetic capacity of the plant [25]. For instance, a high proportion of sucrose/trehalose-6-phosphate indicates a good photosynthetic capacity, whereas a low proportion indicates a low-energy stress [26]. The details of the proteins directly involved in the process remain unclear; one of the most plausible candidates to mediate this energy homeostatic process is the enzyme sucrose nonfermenting-1 related kinase 1 (SnRK1) acting as a sensing hub through phosphorylation signaling of protein targets [27, 28].

Starch is the main nonstructural carbohydrate playing a crucial role as energy storage molecule. It is composed of amylopectin (70–80%) and amylose (20–30%); both polymers are made of D-glucose. Amylose is a linear chain molecule linked by *α*-1,4 bond, whereas amylopectin is a branched polymer linked by *α*-1,6 bond. Starch has a semicrystalline and insoluble structure. It forms superior structures, namely, granules that vary in size and shape (polygonal, spherical, and lenticular), amylose/amylopectin ratio, nanostructure, and crystallinity. Both amylose and amylopectin form 95–99% of the dry weight of starch granules [23, 24].

Cellulose and hemicellulose (a polymer of pentoses, mainly xylose) are the main carbohydrate polymers that form the plant cell walls. In addition, lignin, a polymer of phenolic monomers synthesized from aromatic amino acids, is cross-linked with cellulose and hemicellulose fibrils. The proportions of these three components vary depending on the developmental stage of the plants, organs, and species. The cell wall is the main structure involved in plant growth, weight support, and mechanical protection against pathogens [10, 11].

## 3. Plant Survival and Carbon Distribution

In the course of natural history, plants have evolved sophisticated mechanisms to sense the environment and develop possible strategies for survival. Plant movement is highly restricted owing to their sessile nature; the site where they germinate will be most likely their permanent location and they must thrive with the available resources, abundant or scarce, to obtain energy. In the decision-making process, different variables, such as day length, light quality, temperature, direction of gravity, and internal or external molecules, have to be considered, and then, responses such as germination, energy consumption rate, growth speed, organ architecture, and juvenile/maturity transition are triggered [29, 30].

Starch is the preferred molecule for energy storage in the form of chemical bonds. The homeostasis between its synthesis and consumption is central for plant survival [23, 24]. During the day through photosynthesis, the chloroplast continuously captures electrons in NADPH and restores high-energy phosphate bonds in ATP; both molecules are utilized to fix atmospheric CO_2_ in the carbohydrates. Sugars derived from photosynthesis can be used as monomers for structural polysaccharide synthesis that sustain biomass accumulation (cellulose and hemicellulose), employed to transfer energy to the mitochondria for primary metabolism or stored as starch in the chloroplast [31].

Current research using the model plant* Arabidopsis thaliana* indicates that wild plants prefer conservative strategies for energy homeostasis management [32, 33]. The flux and distribution of carbon from the atmosphere to starch are pivotal to assure plant survival during the night until dawn [34]. If not estimated correctly, plants can face severe starvation symptoms with a negative impact on productivity [35]. This control is achieved through an elaborated network of interconnected mechanisms at different levels of the genetic flow of information, for example, circadian expression control to achieve precise expression of catabolic (day) and anabolic (night) enzymes [36], allosteric enzymatic regulation to allow a rapid response to products and other pathways [37, 38], source-sink relations to optimize long-distance sugar transport and regulate growth [31], autophagy as a low-energy stress response or nutrient recycling from senescing leaves [39], nutrient mobilization that involves other important metabolites like amino acids [40], and molecular signaling to sense metabolic strength at the cell and whole organism level [41].

Only when accumulation of resources and construction of structures indicate the existence of robust photosynthetic machinery, the plant prioritizes in allocating its resources to meet the energy demands of the reproductive stages [32]. In extreme cases, such as drought or delayed development, when the accumulation of resources is unavailable, the plant focuses on the reproductive stage under suboptimal conditions, risking offspring success rather than not having any [25].

## 4. Carbon Allocation and Biotechnology

For the past millennia, plant domestication formed the basis of modern agriculture to sustain human nutrition. It was achieved through the selection of plant individuals with a modified response on one or more of the above-mentioned carbon allocation strategies. For example, modern maize plants concentrate photosynthates on a few reproductive units instead of many, as is performed by its wild-type relative [42]. Wheat cultivars obtained during the Green Revolution have a smaller vegetative biomass capable of sustaining the reproductive stages [43]. Sugarcane has substituted starch for sucrose as its primary storage molecule [44]. Some bean varieties are insensitive to photoperiodic control of flowering [45]. All these achievements in plant domestication modified the natural strategies used to conserve and allocate carbon resources [43, 46, 47].

The strategies employed by domesticated plants have posed high risk for energy and carbon management in the wild, as they are only successful in the fields due to modern agricultural practices overseen by humans. A few examples of the latter are the selection of planting seasons, spacing among individual plants, full assurance of nutritional needs, limiting herbivores and pathogens, and removal of sunlight competitors and debris from the previous generation. Through these strategies, natural hazards that inhibited daring traits in wild plants during natural evolution are now artificially controlled.

New plant phenotypes are needed to adapt to modern challenges such as the increasing human population or the effects of global warming: droughts, floods, and new predator ranges. To address this issue, plants can be modified to invest more energy on perception and protection mechanisms that were rare, less abrupt, or tolerable in nature. Some successful strategies are commercial hybrids transformed with bacterial RNA chaperones (Droughtgard®; [48]) or the SUB1 nontransgenic rice lines that can survive submergence for one week more than the traditional cultivars [49]. Recently, promising increases in plant biomass productivity have been reported by modifying the highly conservative mechanisms of photoprotection in plants [50]. Mickelbart et al. [51] reviewed other successful examples applied in edible crops including multiple copy genes, ecotype and cultivar screening, and the potential use of precision genome editing by CRISPR-Cas9 or transcription activator-like effector nucleases (TALEN). These two latter methods allow introducing DNA sequence changes on specific chromosomal sites; both rely on DNA cleavage by nucleases and subsequent strand repair by natural mechanisms [52, 53] and can be enhanced by adding guide oligonucleotides to increase efficiency and specificity [54]. In plants, deletions, substitutions, and insertions ranging from single-base up to chromosomal rearrangements have been reported [55]. These working strategies can be currently applied to improve plant biomass for the bioethanol industry. Although institutions and companies have produced dozens of edited crops, most applications are directed towards solutions for the food industry and pathogen control [55]. Only in sugarcane, TALEN has been explicitly employed to increase biomass quality for the bioethanol industry [56, further discussed in the next section].

Carbon distribution in bioethanol cultivars should be allocated in carbohydrates and must be readily made available by saccharification for the subsequent fermentation [10, 43]. There are already traditional crops with carbon allocation strategies favoring starch or free sugar accumulation on specific plant tissues, such as maize and sugarcane; ethanol produced from this type of biomass is called first-generation bioethanol [57]. However, some sectors of society are concerned about this technological possibility, since sugarcane and maize are also sources of elemental edible products, and a “food versus fuel” controversy rises that involves competition in land use and impact in future food prices [58]. To resolve this complication, biomass of agricultural residues or whole plants that are not used for human consumption, such as grasses and trees, and that already grow in nonagricultural land can be conserved, managed, and utilized [10, 59, 60]. The ethanol produced from these species is called second-generation bioethanol.

Different strategies have been implemented to identify novel carbon accumulation and distribution patterns and improve saccharification traits in plants intended for bioethanol production, such as the constitutive or temporal inhibition of starch-degrading enzymes [61], delay of flowering time with transcription factors [62], endogenous expression of cell wall degrading enzymes [63], and silencing of lignin biosynthetic enzymes [64].

In some cases, altering carbon distribution on crucial storage or architectural molecules in such radical ways negatively impacts developmental goals and basic growth with the consequence of a penalty in biomass production. Suboptimal mechanics and phasing out of nutrient/development relations should be avoided. To resolve these challenges in the manipulation of carbon allocation to explicitly improve plant traits of interest for the bioethanol industry, novel strategies are needed for fine-tuning carbon fluxes, avoiding negative impacts on critical developmental stages, compromising tissue integrity, and overexposing the plant to pathogen risks. Naturally, plants already have insurance mechanisms for these setbacks, for example, those expressed during cases of low oxygen exposure [40], before dawn [30, 35], and fluctuating light [50]. The understanding and characterization of these pathways can lead to innovative applications in the biotechnology of carbon conservation and allocation.

## 5. Biotechnological Strategies to Increase Plant Biomass Saccharification

With all the current information on carbon accumulation management in plants, different strategies have been tested to improve plant saccharification and, in consequence, bioethanol yield. Currently, they can be classified by the manipulation of timing/location of transcription factors (TFs) and enzymes to achieve one or more of the following objectives: delay of flowering time, starch conservation, and decrease saccharification recalcitrance of the cell wall molecular components. In addition, the use of plant genetic diversity is also explored to discover new genetic factors with positive impact on these applications. In the following sections, we discuss paradigmatic examples of these strategies and current research that, in our perspective, is moving forward the knowledge on this biotechnological field.

### 5.1. Transcription Factors

**Figure 1:**
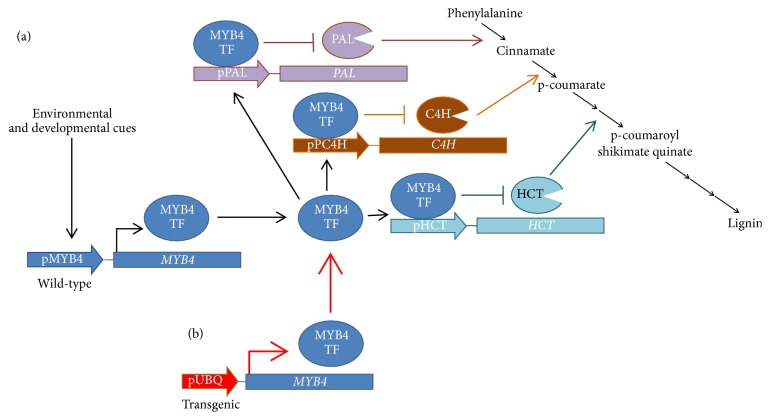
Use of MYB transcription factors to decrease cell wall recalcitrance and increase saccharification yield. (a) Wild-type plants have a gene coding for MYB4, a transcription factor inhibiting several biosynthetic enzymes of lignin under the control of environmental and developmental cues. (b) When MYB4 is expressed using a constitutive strong promoter, it decreases the synthesis of lignin and, in consequence, its recalcitrance to saccharification [12]. PAL: *PHENYLALANINE AMMONIA LYASE*; C4H: *CINNAMATE 4-HYDROXYLASE*; HCT: *HYDROXYCINNAMOYLTRANSFERASE*.

TFs are proteins that can reversibly bind to DNA and simultaneously promote and/or inhibit the expression of multiple genes. In this view, some TFs have been discovered to control several steps of lignin biosynthesis, for example, the family of MYB proteins [65]. When the* MYB4* gene, a partial transcriptional inhibitor of lignin biosynthetic genes was expressed in switchgrass* (Panicum virgatum) *without the control of its native promoter but under the control of a strong constitutive promoter, a decrease in biomass recalcitrance to saccharification was observed (Figure 1). This grass has great potential as a bioenergy crop. In this same report, the importance of testing different transgenic lines of the same construct was highlighted, because the site where the transgene is inserted will affect the expression of the gene of interest. The authors observed that lines strongly expressing the transgene did not survive in field conditions [12].

The use of constitutively expressed MYBs was also tested in sugarcane. Two TFs were analyzed to determine which one could simultaneously inhibit more cell wall biosynthetic genes. Interestingly, it was also found that one MYB could increase free sucrose [66].

**Figure 2:**
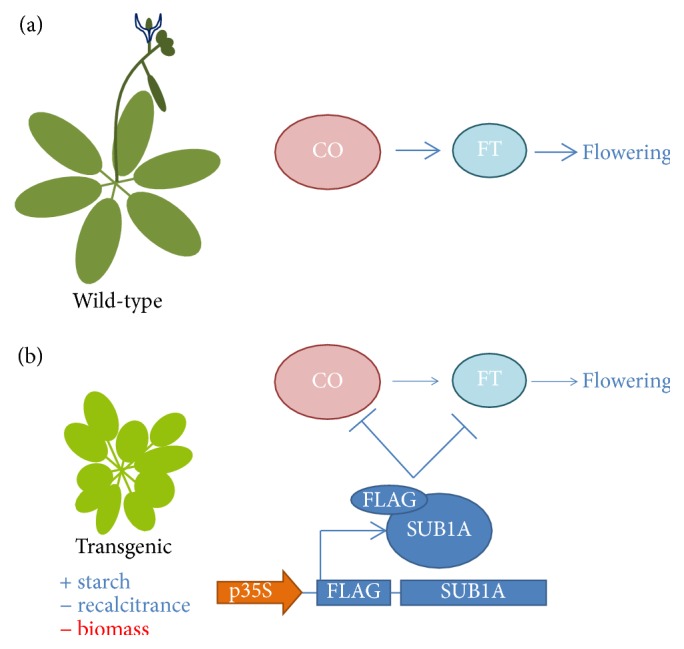
Increase in starch concentration by using rice SUB1A-1 transcription factor. (a) Wild-type plants have an intact flowering signaling system through *CONSTANS (CO)* and* FLOWERING LOCUS T (FT)*. (b) Plants transformed with the *ETHYLENE RESPONSE FACTOR *gene* SUBMERGENCE1A-1 (SUB1A)* have a delay in flowering time through the transcriptional inhibition of* CO *and* FT*, temporally accumulating starch otherwise used to fuel flowering development [13].

Another family of TFs that have been used to improve plant biomass saccharification is the* ETHYLENE RESPONSE FACTORS (ERFs)*. Núñez-López et al. [13] explored the capacity of two ERFs naturally involved in the plant response to flooding stress, an energy limiting stress, and found that* SUB1A-1 *overexpression produced a phenotype where starch conservation was doubled, especially in preflowering stages (Figure 2). It was hypothesized that SUB1A-1 caused this effect through the associated effect of flowering time inhibition; SUB1A-1 strongly repressed the expression of classic flowering genes *CONSTANS *and* FLOWERING LOCUS T* [67]. The cell wall resistance to deformation was also decreased. On the other hand, the side effect of this strategy was lower biomass accumulation after flowering, highlighting the importance of testing new expression patterns in time and space. Wuddineh et al. [68] performed a wide screening of switchgrass ERFs to find a suitable candidate for the expression in young and expanding tissues. In this way,* ERF001* was selected and its overexpression increased biomass nearly double of that of wild-type plants.

Flowering has been also manipulated with TFs of the *SQUAMOSA PROMOTER BINDING PROTEIN-LIKE (SPL)* family. It was found that* SPLs *participate as a last resort flowering mechanism activated by the age of the plant and is naturally repressed in young plants by miRNA156 [25]. In this manner, when miRNA156 was more expressed in natural mutants or genetically engineered plants, the juvenile phase was extended, and, interestingly, starch and cell wall saccharification were increased [62]; this constitutes one of the few examples where these two highly desired characteristics in plant biomass for biofuels are simultaneously improved.

TFs of the ZIP family have been used to improve sugar content in tomato fruits. It was observed that these proteins are under the control of upstream open reading frame (uORF) domains capable of responding to the cellular concentration of sucrose. When uORFs were removed and the ZIP genes were expressed from fruit-specific promoters, both glucose and fructose concentrations were increased in tomato [69]. This innovative approach can be extended to engineer ZIP TFs for the bioethanol industry.

### 5.2. Enzymes

**Figure 3:**
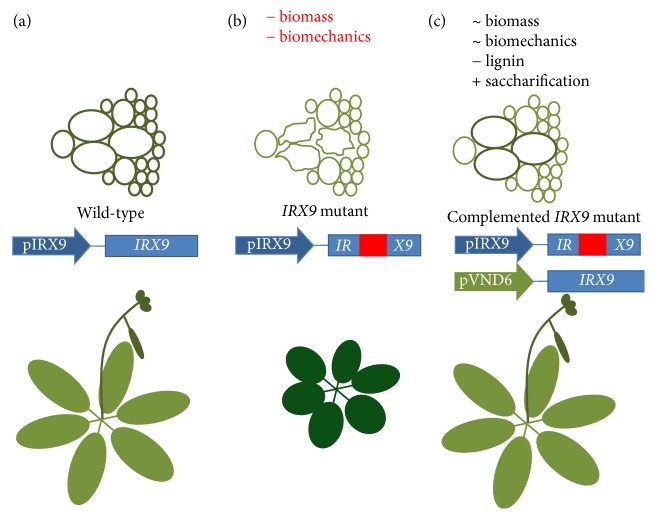
Increase in cell wall saccharification by using loss-of-function genetic backgrounds and complementation by tissue-specific expression. (a) Wild-type plants have cell walls with high lignin content, optimal biomechanical properties, and recalcitrance to saccharification. (b) Loss-of-function mutants (T-DNA knockout, red insertion) of glucosyltransferase* IRX9* have collapsed vascular cells with suboptimal biomechanical properties. (c) The expression of* IRX9* over the mutant background under the control of a xylem-specific promoter rescues the biomechanical properties and leaves the rest of the cells with low lignin content, less recalcitrance, and better saccharification [14].

Different enzymes have been tested as biotechnological tools to improve saccharification. Using the information from cell wall architecture mutants of* Arabidopsis*, Biswal et al. [70] discovered that different glucosyltransferases (GTs) participate in several lignin biosynthetic steps. To improve the saccharification of* Populus deltoides*, a cold-weather tree with desirable bioenergy traits at the juvenile stage, the selective gene silencing of GTs was tested. It was observed that lignin content did not change in these mutants; however, the chemical proportion of its components was modified, and biomass and saccharification increased up to 38% and 10%, respectively. A related application is the use of loss-of-function mutants of *IRREGULAR XYLEM 9 (IRX9)*, a GT coding gene, and its expression from a xylem-specific promoter (Figure 3); this augmented up to 30% saccharification of the cell wall [14].

*CAFFEOYL SHIKIMATE ESTERASE (CSE)* is a gene coding for an enzyme involved in lignin biosynthesis. The* cse *loss-of-function mutants have increased cell wall saccharification up to 300% but suffered severe biomechanical defects in the plant vascular system [71]. To resolve this drawback, Vargas et al. [72] expressed* CSE *under the control of vascular system promoters, the most collapsed tissue in* cse *mutants. This approach repaired plant vasculature, normalized development, and retained the trait of improved saccharification.

Another gene coding for a lignin biosynthetic enzyme that has been used in saccharification biotechnology is *CAFFEIC ACID O-METHYLTRANSFERASE (COMT)*. To avoid unwanted plant phenotypes, a gene silencing strategy was tested where gene expression is not fully suppressed but only decreased. Plants with silenced* COMT *did not have increased pathogen susceptibility or biomechanical defects, but lignin content was decreased, and dry matter and saccharification yield were increased with a total improvement of bioethanol yield up to 25% when compared to that in wild-type plants [73]. These data were obtained in field trials, a remarkable feature of this research since few saccharification improvement strategies have been tested at this level [12, 73]. Recently, this strategy has been implemented in sugarcane through TALEN-mediated multiallelic knockout mutagenesis of* COMT* with similar results [56], constituting the first example of precise genome editing for improvement of plant biomass quality.

**Figure 4:**
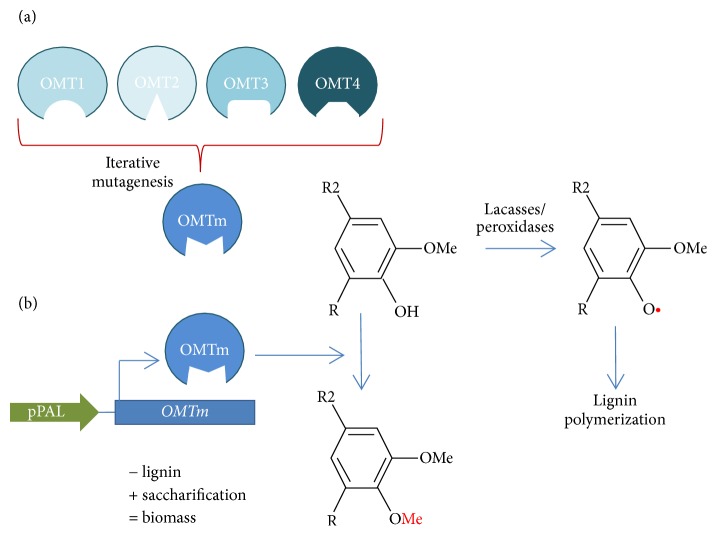
Reduction in lignin polymerization and increase in saccharification through protein engineering of O-methyltransferases (OMTs). (a) Using computational studies, the amino acids in the active site of the enzyme monolignol 4-O-methyltransferase (OMT) are identified and subjected to iterative mutagenesis to obtain a mutated enzyme (OMTm). (b) The gene coding for OMTm is expressed* in planta *from a specific promoter of expanding cells (pPAL). The OMTm methylates the phenolic moieties of lignin inhibiting further polymerization [15]. pPAL: promoter of *PHENYLALANINE AMMONIA LYASE*.

Protein engineering is an innovative biotechnological tool recently employed to modulate lignin synthesis. Cai et al. [15] inhibited lignin polymerization by expressing the enzyme monolignol 4-O-methyltransferase (OMT) in* Populus*. The sequences of the OMTs used were artificial variants obtained by iterative saturation mutagenesis of multiple plant OMTs with different catalytic capacities and activities [74]. This method allowed testing the effect of amino acid substitution in the active site to obtain novel activities, in this case, methylation of phenolic compounds to inhibit their use as polymerization substrates (Figure 4). Modified plants with engineered OMTs yielded up to 40% more ethanol than wild-type plants [15]. Further, using protein engineering, Yang et al. [75] screened and constructed chimeric proteins combining carbohydrate binding domains and iron binding domains. In this way, the chimeric protein captured iron and concentrated it* in vivo* in the cell wall where it acted as an inorganic catalyst for saccharification.

Another alternative explored is the enzymatic control of cell expansion. As previously mentioned, plants have carbon allocation strategies that can be considered conservative, that is, do not express their full potential to enhance growth in order to maintain abundant energy reserves when faced with unexpected stress. Gibberellic acid (GA) is a phytohormone that mainly regulates cell elongation in plants and, in consequence, carbon and energy commitment in the processes [32]. With this knowledge, Do et al. [76] constitutively overexpressed the maize gene *gibberellin 20-oxidase (GA20Ox)*, coding for a GA biosynthetic enzyme, in the bioenergy grass* P. virgatum*. Plants doubled in dry weight. When a homolog of this gene was expressed using a xylem-specific promoter, stem biomass was tripled in* Populus *[77]. In both of these reports, the authors did not test the effect on biomass recalcitrance to saccharification; however, it might be expected that the increases in biomass will improve ethanol yield of these cultivars.

A novel approach is to search for other plant carbohydrate polymers with low recalcitrance to saccharification. One option is mixed-linkage glucan (MLG); with low secondary structure complexity, it is easily subjected to saccharification by commercial enzyme mixtures. However, accumulation in the plant cell causes severe developmental impacts. To sort this disadvantage, Vega-Sánchez et al. [78] expressed in* Arabidopsis *the MLG biosynthetic gene *CELLULOSE SYNTHASE-LIKE F* (CSLF4) using a senescence-specific promoter; transformed plants had a normal transition through developmental stages and showed improved saccharification at the late stages. This strategy would allow the use of senescing biomass that usually remains as agricultural waste in the fields.

### 5.3. Natural Genetic Diversity and Mutagenesis

One classical tool of agronomic science that can be used in bioenergy crop research is the screening of induced or natural diversity for interesting traits. Li et al. [79] induced genetic diversity in rice by chemical (EMS) or biological mutagenesis (T-DNA) and found mutants in elite genetic backgrounds with higher cell wall saccharification; this opens the possibility of using nonedible organs for bioethanol production. Using the grass model plant* Brachypodium distachyon*, Marriott et al. [80] created a chemically (azide) mutagenized population and isolated a set of mutants with higher saccharification yields ranging from 20 to 60% more than wild-type plants; interestingly, mutants with low saccharification yields were also isolated, providing genetic material for experiments aimed to discover new biochemical routes for the industry. Finally, Stamatiou et al. [81] designed a mass screening of known and new* Arabidopsis *mutants and isolated a set of phenotypes with increased saccharification caused by defects on starch-degrading enzymes, modified auxin transport, and other mechanisms yet to be identified.

Microalgae are also photosynthetic organisms and their use in the bioenergy industry has been focused on biodiesel raw material owing to their high lipid content. However, recent studies pointed out that, in the microalgae genetic diversity, different carbon accumulation mechanisms that differ from those of land plants and that may be of future interest in ethanol biotechnology exist [60, 82].

The natural diversity of cell wall self-deconstruction mechanisms expressed by plants should also be of interest in technology development, for example, aerenchyma formation during submergence, the remobilization of nutrients from senescing leaves and their abscission, and cell wall expansion during fruit maturation and organ growth. In these activities, new enzymes capable of degrading different plant polymers can be found [83].

Another aspect of natural genetic diversity is the search of genetic promoters capable of directing the expression of proteins of interest with innovative patterns. This idea has already proved its value in this area of biotechnology (see* Enzymes*). The expansion of available promoters will enrich the genetic toolbox to design cultivars with different saccharification contexts on tissue, development, and environmental levels. Ko et al. [84] performed genome-wide microarray hybridization with different tissues of* Populus *to create a catalog of promoters and its patterns of interest in the saccharification field. In the bioenergy grass* Arundo donax*, studies aiming to characterize tissue-specific expression for genetic improvement of saccharification traits have been performed [85].

To study genetic diversity in sugarcane, a research was done with its evolutionary ancestors. Using histochemistry, cell wall analysis, and saccharification test, De Carli et al. [86] found that recalcitrance is a multigene characteristic and is not homogenous among tissues. Another characteristic that has not been studied using molecular tools but is of interest in the bioethanol industry is lodging resistance, an undesirable trait in large grasses grown in the tropics. Rueda et al. [87] performed a screening of cultivars of the bioenergy grass* Cenchrus purpureus* in field conditions and found contrasting genetic diversity in lodging resistance. These genetic backgrounds should be adequate to uncover the molecular basis of this phenomenon to significantly improve plant quality as raw material for the bioenergy industry.

## 6. Conclusions

Transition from our current economy with high hydrocarbon consumption to a future with low environmental impact requires the development of new technologies such as ethanol biofuels. The components of plant biomass such as starch, cellulose, hemicellulose, and other carbohydrate polymers directly impact the quality required for this industry to succeed. Understanding the different genetic factors (enzymes, TFs, and promoters) that control the anabolism and catabolism of plant carbohydrate polymers is the first step toward the development of biotechnologies. The strategies reviewed herein are based on the molecular manipulation of carbon distribution in plants, and its proof-of-concept has demonstrated successful in model plants. Some of this knowledge has been tested in plants of industrial significance and under field conditions. Nevertheless, there is a challenge to transition through all phases of technology development. Another step is to consolidate plant research in the tropics, especially cultivars of sorghum, sugarcane, and nonedible warm weather grasses. All these innovative biotechnological examples are of interest not only for the bioethanol industry, but also for the improvement of species used for human nutrition. Both can be combined to concomitantly satisfy the growing demand of plant raw materials for feeding and energy purposes.
